# Diagnostic performance and clinical utility of shear-wave elastography in musculoskeletal soft-tissue tumors: a systematic review

**DOI:** 10.3389/fonc.2026.1768309

**Published:** 2026-06-30

**Authors:** Yayun Lin, Jianping Wang, Hao Sun, Jianmin Zhou

**Affiliations:** 1 Department of Ultrasound, Yantaishan Hospital, Yantai, Shandong, China; 2 Department of Ultrasound, Sinopharm Tongmei General Hospital, Datong, Shanxi, China

**Keywords:** diagnostic accuracy, magnetic resonance imaging, musculoskeletal ultrasound, reproducibility, shear-wave elastography, soft-tissue tumors

## Abstract

**Background:**

Shear-wave elastography (SWE) is increasingly used to assess musculoskeletal soft-tissue masses, but its diagnostic performance relative to ultrasonography (US) and magnetic resonance imaging (MRI) remains uncertain.

**Objective:**

To evaluate the diagnostic accuracy, reproducibility, and clinical utility of SWE for differentiating benign and malignant soft-tissue tumors.

**Methods:**

Following PRISMA-DTA guidelines, PubMed/MEDLINE, Embase, Web of Science, Scopus, ProQuest Dissertations and Theses, and RSNA/WFUMB proceedings were searched through 22 September 2025. Eligible studies included patients with histologically verified extraosseous soft-tissue masses evaluated using quantitative SWE. Sensitivity, specificity, and area under the ROC curve (AUC) were extracted or reconstructed. Owing to marked methodological and threshold heterogeneity, a narrative synthesis was performed. Certainty of evidence was rated with GRADE-DTA.

**Results:**

Ten studies (n = 1,335 lesions; 22-41% malignant) met inclusion criteria following a search of six databases (PubMed/MEDLINE, Embase, Web of Science, Scopus, ProQuest Dissertations and Theses, and RSNA/WFUMB proceedings) through 22 September 2025. SWE reproducibility was high where reported (intraclass correlation coefficient > 0.85 in four studies), but diagnostic performance varied widely (AUC range 0.57-0.87). In some studies, In selected studies, SWE was associated with potentially higher specificity in benign-appearing or superficial lesions (ΔAUC +0.07 vs US in one cohort), but it failed to distinguish lipomatous or deep lesions from malignancy. MRI remained superior in most direct comparisons (AUC 0.85-0.90). Threshold heterogeneity and poor cross-vendor reproducibility limited generalizability. Certainty of evidence was moderate for superficial lesions and low for deep or lipomatous tumors.

**Conclusion:**

SWE demonstrates excellent reproducibility but inconsistent diagnostic utility across lesion types. It should be applied selectively primarily for superficial, non-fatty lesions as an adjunct to US rather than as a stand-alone diagnostic tool. Standardized acquisition protocols and vendor calibration are prerequisites for broader clinical adoption.

**Systematic review registration:**

https://www.crd.york.ac.uk/prospero/display_record.php?RecordID=1172017, identifier CRD420251172017.

## Introduction

Soft-tissue tumors range from common benign lesions such as lipomas to rare but clinically significant malignancies known as soft-tissue sarcomas (STS). Although benign masses predominate, STS are aggressive and often detected late. Contemporary global estimates suggest an incidence of approximately 1–5 per 100,000 annually, reinforcing the need for accurate differentiation at presentation (1–4). Benign lesions including lipomas, which affect 1-2% of the population, vastly outnumber sarcomas, yet misclassification can lead to delayed oncologic care or unnecessary biopsies and surgery (5–9). Ultrasound (US) is widely used as the first-line modality for evaluating soft-tissue masses due to its accessibility, lack of ionizing radiation, and ability to correlate imaging findings with palpable abnormalities (10). Conventional B-mode and Doppler imaging provide important morphologic and vascular information, but there remains substantial overlap in sonographic features of benign and malignant lesions. MRI offers superior anatomic delineation but has limited availability, higher cost, and imperfect specificity for malignancy (3).

Shear-wave elastography (SWE) is a quantitative ultrasound (US) technique that measures tissue stiffness by calculating the speed of propagated shear waves, expressed as shear-wave velocity (m/s) or Young’s modulus (kPa). Malignant tumors commonly exhibit increased cellularity, stromal disorganization, and fibrosis, resulting in higher stiffness compared with benign masses. SWE is less operator-dependent and more reproducible than strain elastography because it does not require manual compression (11–15). However, a critical technical challenge in the musculoskeletal (MSK) setting is tissue anisotropy: unlike parenchymal organs such as the liver, skeletal muscle and connective-tissue tumors display direction-dependent stiffness that varies with probe orientation. This anisotropy, combined with viscoelastic properties of MSK tissues, means that universal kPa thresholds cannot be extrapolated from non-MSK organs and explains why no single cut-off has proven generalizable across studies.

Multiple prospective studies have evaluated SWE for distinguishing benign from malignant musculoskeletal (MSK) soft-tissue lesions using histopathology as the reference standard. Findings have been mixed: some cohorts reported improved diagnostic classification when SWE was combined with conventional US despite no universal stiffness threshold (16, 17), while others found that specific SWE parameters particularly elasticity heterogeneity (e.g., SD) were independently associated with malignancy (18). A sarcoma-board study identified a tumor-to-fat elasticity ratio that achieved high specificity but insufficient sensitivity to replace biopsy (19). Collectively, these studies highlight heterogeneity in platforms, acquisition protocols, and lesion characteristics, suggesting that SWE may be most useful as an adjunct rather than a standalone test. At the evidence-synthesis level, a prior systematic review reported “relatively good” pooled diagnostic performance but substantial heterogeneity related to test thresholds, case mix, and device variation (20). Importantly, the evidence base has evolved considerably since 2021: at least four new prospective studies (19, 21–23) have reported divergent findings, including a large multi-vendor comparison (23; n = 269) that fundamentally challenged prior conclusions on cross-platform generalizability. Emerging techniques including point-SWE/ARFI, superb microvascular imaging (SMI)-combined protocols, and heterogeneity-based metrics (E_SD) have also been evaluated, none of which were covered by Wu et al. Taken together, these developments necessitate an updated synthesis incorporating subgroup analyses by lesion depth, composition, and platform.

This systematic review aimed to evaluate the diagnostic accuracy, reproducibility, and clinical utility of shear-wave elastography (SWE) for differentiating benign and malignant soft-tissue tumors using histopathology as the reference standard. Specifically, we synthesized evidence on (i) sensitivity, specificity, and AUC; (ii) variation in performance by lesion depth, size, and histologic subtype; and (iii) the influence of vendor platform and threshold heterogeneity, as well as integration with other imaging modalities.

## Methods

### Design and registration

This systematic review followed PRISMA 2020 and PRISMA-DTA reporting guidelines. The protocol was developed *a priori* and registered in PROSPERO (CRD420251172017). Pilot searches refined key concepts and controlled vocabulary. Full methods, including expanded search logic and SWE-biopsy interval rationale, are provided in **Supplementary Appendix 1**. The final search covered all literature published or indexed through 22 September 2025.

### Eligibility criteria

**Figure 1:**
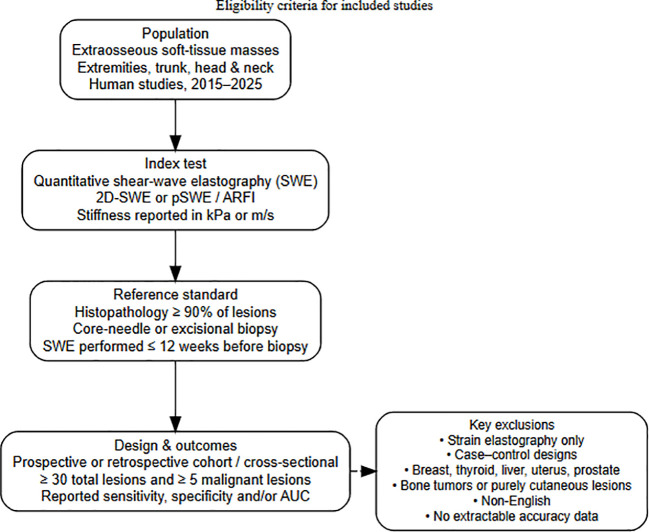
Flow diagram summarizing the eligibility criteria used to identify studies for inclusion in this systematic review.

Studies were eligible if they evaluated quantitative shear-wave elastography (SWE) for differentiating benign and malignant extraosseous soft-tissue masses of the extremities, trunk, or head and neck. Prospective or retrospective cohort and cross-sectional designs were included. Acceptable SWE methods were 2D-SWE and point-SWE/ARFI reporting stiffness in m/s or kPa. Studies of strain elastography alone or case-control designs were excluded to avoid overestimation of diagnostic accuracy (**Figure 1**).

To ensure adequate precision, eligible studies required ≥30 lesions overall, ≥5 malignant cases, and ≥90% histologic verification by core-needle or excisional biopsy. Parenchymal organs (e.g., breast, thyroid, liver, uterus, prostate), bone tumors, and purely cutaneous lesions were excluded. SWE examinations had to occur within 12 weeks of histologic verification to reduce temporal bias (detailed justification in **Supplementary Appendix 1**). Studies relying solely on imaging or clinical follow-up were excluded from primary analyses but retained for sensitivity considerations.

### Reference standard

Histopathology served as the reference standard. Studies using histology for malignant/suspicious masses and follow-up only for presumed benign lesions were classified as high risk for partial verification bias and summarized narratively. No study without histopathologic confirmation of malignancy was included in the main synthesis.

### Index test framework

Included studies examined SWE either as a standalone diagnostic test or as part of a multiparametric ultrasound assessment with B-mode and/or Doppler. Primary analyses focused on SWE alone; secondary analyses described its incremental diagnostic contribution relative to conventional ultrasound.

### Outcomes

Primary outcomes were sensitivity, specificity, and area under the ROC curve (AUC). Extracted SWE metrics included absolute stiffness values and relative indices (e.g., tumor-to-fat ratios). When multiple thresholds were reported, the value with the highest Youden index was used for comparisons; alternative thresholds were tabulated. Secondary outcomes included reproducibility measures (e.g., intraclass correlation coefficients). Indeterminate or technically inadequate SWE studies were retained for sensitivity description.

### Information sources and search strategy

We searched PubMed/MEDLINE, Embase, Web of Science, Scopus, ProQuest Dissertations and Theses, and RSNA/WFUMB proceedings for human studies published from 2015-2025. Searches were restricted to English-language publications. The PubMed strategy is provided in **Supplementary Appendix 1**. Reference lists of included studies were manually screened.

### Conference abstracts and incomplete data

Conference abstracts were screened but included only when sufficient numerical diagnostic data were reported or when authors subsequently provided missing information. Four included studies had incomplete diagnostic reporting; all corresponding authors were contacted twice over three weeks. Two supplied additional data enabling reconstruction of 2×2 tables; the remaining two were included descriptively.

### Study selection and data extraction

Two reviewers screened all records independently in Covidence and resolved disagreements by consensus. Data extraction captured study design, sample size, lesion characteristics, SWE parameters, thresholds, reproducibility metrics, and diagnostic outcomes. Ambiguities were clarified through author contact when possible.

### Risk of bias assessment

Two reviewers independently assessed each study using QUADAS-2, focusing on threshold pre-specification, blinding, verification bias, and the SWE-histology interval. Studies with ≥90% histologic verification and SWE-biopsy intervals ≤12 weeks were judged low risk. Applicability concerns, including enriched referral cohorts or pediatric-only populations, were documented.

### Data synthesis and analysis

Substantial heterogeneity was anticipated across SWE platforms, acquisition protocols, stiffness metrics, and threshold definitions. As prespecified, a bivariate random-effects meta-analysis was planned, but quantitative pooling was not feasible because:

1. Key 2×2 data were unavailable for several studies even after author contact;
2. SWE thresholds, units, and acquisition parameters varied widely;
3. Multiple studies reported AUC or summary measures only, without extractable sensitivity/specificity pairs.

For transparency, attempted pooling, heterogeneity indices, and model non-convergence outputs are shown in **Supplementary Appendix 3**. Briefly, the bivariate random-effects model failed to converge in Stata (metandi) due to an unidentifiable between-study correlation matrix arising from (i) extreme threshold heterogeneity precluding a common operating point, (ii) five studies reporting only AUC without extractable sensitivity-specificity pairs, and (iii) fewer than six studies providing complete 2×2 tables—below the minimum generally required for stable HSROC estimation. In the studies for which sensitivity and specificity pairs could be extracted (n = 5), the Spearman correlation between logit(sensitivity) and logit(1−specificity) was ρ = 0.43 (p = 0.21), indicating moderate but non-significant threshold effects; the estimated I² for sensitivity and specificity in univariate random-effects models was 87% and 91%, respectively, confirming extreme statistical heterogeneity that further contraindicated pooling.

Accordingly, we conducted a narrative and descriptive synthesis. Study-level sensitivity, specificity, and AUC were summarized using medians and interquartile ranges. Heterogeneity was visualized via scatterplots and forest-style diagrams generated in R (ggplot2, mada). Subgroup trends were described where ≥3 studies provided comparable data, including analyses by lesion depth (8 studies), lipomatous vs non-lipomatous lesions (6 studies), and elasticity heterogeneity metrics (5 studies). Vendor-specific subgrouping was not possible because no SWE platform had ≥3 independent studies.

Threshold heterogeneity was assessed using Spearman correlation between logit(sensitivity) and logit(1 − specificity); a moderate, nonsignificant correlation was observed (ρ = 0.43; p = 0.21). Planned sensitivity analyses for verification bias could not be performed due to lack of individual-level reclassification data; however, indeterminate SWE outcomes were retained descriptively.

All analyses were conducted in Stata 18 (metandi, midas) for planned pooling attempts and R 4.3.0 for final descriptive figures.

### Certainty of evidence

Certainty was assessed using the GRADE-DTA approach. Two reviewers independently evaluated five domains, risk of bias, indirectness, inconsistency, imprecision, and publication bias, with disagreements resolved by discussion. Because no pooled estimates were generated, the imprecision domain was judged using pre-specified surrogate criteria adapted using pre-specified pragmatic criteria for narrative DTA synthesis: (i) total sample size below the information-size threshold for the outcome of interest (operationalized as n < 2,000 for a DTA review with prevalence 22-41% and AUC variability > 0.15); (ii) the range of study-level sensitivity or specificity spanning > 20 percentage points without an assignable methodological cause; and (iii) fewer than three studies providing complete 2×2 data within a subgroup. Certainty was downgraded for imprecision when at least two of these three criteria were met. A qualitative Summary of Findings table presents certainty ratings for superficial, deep, lipomatous, and mixed soft-tissue lesions.

## Results

**Figure 2:**
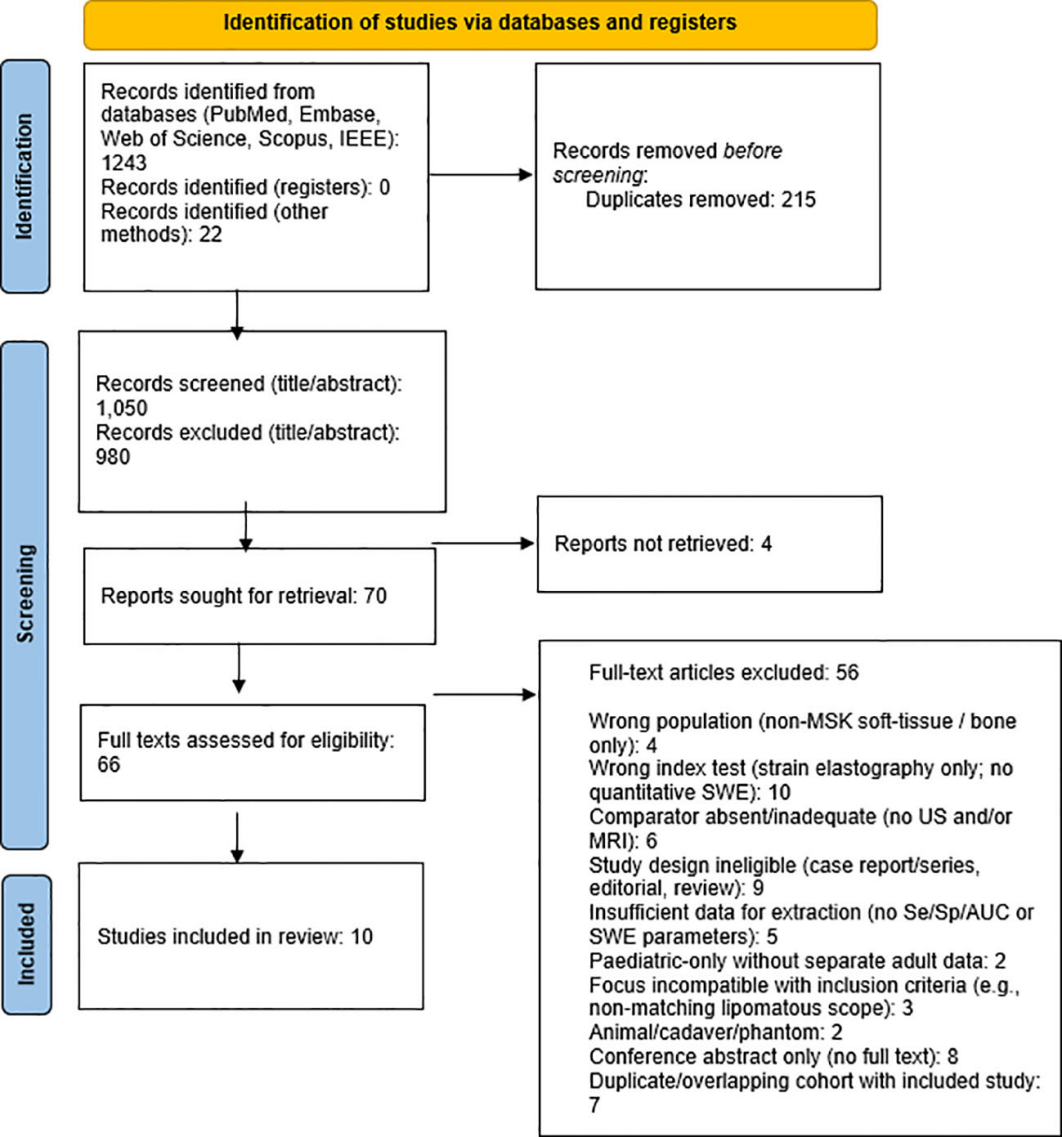
PRISMA flow diagram summarizing study selection for the systematic review.

Ten studies (16–19, 24 (a); 21–25 (b)) involving 1,335 soft-tissue lesions of which 22% to 41% were malignant met the inclusion criteria (**Figure 1**; study selection is illustrated in **Figure 2**). Sample sizes ranged from 50 to 269 lesions, with malignant case counts ranging from 14 to 61 per study. Eight studies were prospective cohorts, one was a retrospective consecutive series, and one was a prospective reproducibility study. Nine studies evaluated SWE alongside conventional ultrasonography (US), and six included magnetic resonance imaging (MRI) as a comparator. SWE modalities included 2D-SWE (n=6), point-SWE/ARFI-based SWE (n=4), and combinations thereof. Studies were conducted in sarcoma referral centers (n=5), tertiary MSK oncology centers (n=3), and university hospitals (n=2) across the UK, France, Turkey, China, and Japan. Full study-level characteristics including design, modality combinations, and clinical setting are shown in **Table 1**.

**Table 1:** Study characteristics of included studies (direct and contextual evidence).

Author (year)	N	Malignant %	Modalities	Comparison type	Design	Setting
16	206	38%	US + SWE + MRI	Direct	Prospective cohort	Sarcoma center (Leeds, UK)
17	148	41%	US + SWE + MRI	Direct	Prospective cohort	Tertiary MSK oncology center (Oswestry, UK)
19	136	40%	US + SWE	Direct	Prospective observational	University hospital (sarcoma-board referral, France)
18	81	26%	US + rtSWE (quant & qual)	Direct	Prospective consecutive	General hospital (China)
24 (a)	105	37%	US + ARFI SWE (VTQ)	Direct	Prospective diagnostic accuracy	Specialist sarcoma center (UK)
21	167	28%	US (SMI) + SWE + MRI	Direct	Retrospective consecutive	University hospital (Japan)
22	109	34%	US + pSWE (VTIQ)	Direct	Prospective diagnostic accuracy	Tertiary university hospital (Turkey)
23	269	22%	US + SWE ± MRI	Direct	Prospective diagnostic accuracy	Sarcoma referral center (UK likely)
25	64	38%	SWE only	Single-modality (reproducibility)	Prospective reproducibility	Sarcoma referral center (UK)
24 (b)	50	30%	US + ARFI SWE	Single-modality	Prospective pilot	Specialist sarcoma center (UK)

**Figure 3:**
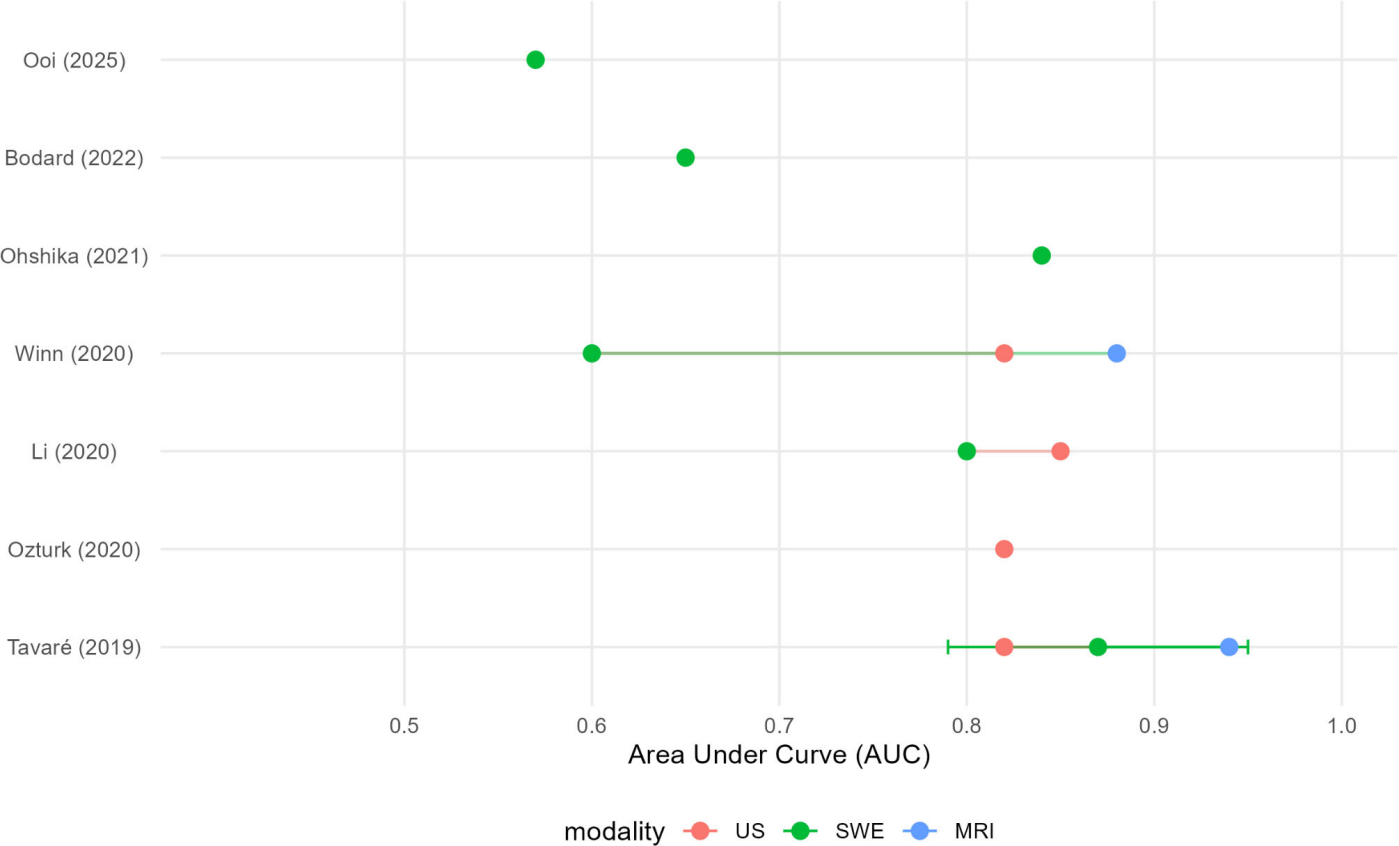
Sensitivity-specificity distribution for ultrasonography (US), shear-wave elastography (SWE), and magnetic resonance imaging (MRI). Scatter plot showing study-level sensitivity and specificity for each modality (US, SWE, MRI).

Across the ten included studies, the diagnostic accuracy of quantitative shear-wave elastography (SWE) showed wide variation (**Table 2**; **Figure 3**). Reported sensitivity values ranged from 50% to 93%, and specificity values ranged from 26% to 85%, with AUC values spanning 0.57 to 0.87 where available. Several studies reported no reproducible or prespecified SWE threshold (e.g., 17, 24a). One study identified an elasticity heterogeneity metric (E_SD > 0.8 m/s) associated with higher classification performance within its cohort (18). In studies where SWE was evaluated alongside other modalities, AUC values for SWE ranged from 0.57 to 0.87, compared with 0.80 to 0.90 for US and MRI when reported.

**Table 2:** Diagnostic accuracy by modality and study.

Author (year)	Modality	N (lesions)	Sensitivity % (95% CI)	Specificity % (95% CI)	AUC (95% CI)	Primary threshold/parameter	Key comparative finding/ΔAUC comment
16	US	206	78 (65-88)	82 (70-91)	0.80	Adler ≥ 2 vascularity	Baseline US reference; SWE ↑ AUC by +0.07 in US-benign subset; MRI AUC 0.89 > SWE.
	SWE	206 (41 benign subset*)	93 (69-99)*	72 (62-79)*	0.87 (0.79-0.95)*	1.78-2.02 m/s (Youden vs 100% Se)	Useful only for US-benign lesions; no value for US-suspicious.
	MRI †	189	92 (81-97)	85 (74-92)	0.89	Enhancement + morphology criteria	MRI superior to US and SWE (+0.12 AUC vs US, p < 0.001).
17	US	148	NR	NR	NR	Expert visual classification	Expert accuracy > logistic model combining SWE + MRI.
	SWE	119 (analyzable subset #)	—	—	—	No reproducible threshold (p = 0.06)	SWE not diagnostic; expert visual > model.
	MRI †	135	83	88	—	Standard protocol (non-standardized)	MRI US performance (AUC not reported).
19	SWE	136	50	79	0.68 (0.52-0.75)	40.8 kPa (Youden)	Specificity-favoring; best in non-fatty lesions.
18	US	81	71	87	0.85	Qualitative pattern III-IV	Baseline US AUC 0.85; rtSWE complementary.
	rtSWE (quant + qual)	81	67	85	0.80	E_SD > 0.8 m/s (Youden)	Slightly lower AUC (Δ -0.05 vs US); E_SD strongest predictor.
24 (a)	US	105	77	79	—	B-mode classification	Baseline for ARFI comparison.
	ARFI-SWE	105 (39 malignant)	—	—	—	Velocity 2.8 m/s (no cut-off)	High measurement stability; no diagnostic difference.
21	SWE alone	167	—	—	0.84	≥ 7.9 m/s (Youden)	SWE alone AUC 0.84; SMI + SWE → AUC 0.90 (Se 94%, Sp 79%).
22	US	109	91.9	72.2	0.82	US vascularity type	US depth/vascularity predicted malignancy.
	pSWE (VTIQ)	109	—	—	—	3.3 vs 3.0 m/s (NS)	SWE not discriminatory.
23	SWE	269	83 (72-91)	26 (18-35)	0.57	1.78-2.02 m/s (pre-specified)	Poor specificity and cross-vendor agreement (ΔAUC 0 vs US 0.82).
25	SWE	64	—	—	—	Reproducibility study (no cut-off)	ICC 0.85-0.92; limits of agreement ± 40%.
24 (b)	US	50	73	77	—	B-mode score	B-mode adequate for triage; pilot feasibility dataset.
	ARFI-SWE	50	—	—	—	No threshold (ICC ≥ 0.9)	Reproducible but not diagnostic.

ARFI, acoustic radiation force impulse; pSWE, point SWE; SMI, superb microvascular imaging; E_SD, elasticity standard deviation; NR, not reported.

* Subgroup of US-benign/probably benign lesions in (16).

When SWE was combined with other imaging parameters, some studies reported higher AUC values than SWE alone (e.g., AUC = 0.90 for SMI + SWE; 21). Conversely, two studies reported minimal separation between benign and malignant lesions using SWE metrics (22, 23). Studies focusing on reproducibility reported high measurement consistency (ICC ≥ 0.85) but did not provide diagnostic cutoffs suitable for classification (24, 25b). Given the prevalence range of 22-41% malignancy across studies, the clinical utility of SWE is best understood through likelihood ratios. Across the studies reporting sufficient data to reconstruct sensitivity and specificity (n = 5), positive likelihood ratios (LR+) ranged from 1.4 to 4.0 and negative likelihood ratios (LR−) ranged from 0.19 to 0.72. The highest LR+ (∼4.0; 16, benign subset) indicates a moderate post-test probability increase for malignancy when SWE is positive, but is insufficient to confirm malignancy without additional imaging. The most favorable LR− (∼0.19; 21, SMI+SWE combined) suggests that a negative combined result could meaningfully reduce post-test probability in referral settings; however, standalone SWE LR− values were generally > 0.50, providing limited rule-out capacity. These findings reinforce that SWE cannot replace biopsy or MRI as a definitive triage tool.

**Table 3** summarizes direct head-to-head comparisons of shear-wave elastography (SWE) with ultrasonography (US) and magnetic resonance imaging (MRI). Across the seven comparative studies, SWE increased the AUC from 0.80 to 0.87 in US-benign lesions (+0.07) but remained below MRI (0.89). Other studies reported no reproducible SWE threshold (17, 24 [a]) or specificity-heavy profiles with modest sensitivity (19: Se = 50%, Sp = 79%, AUC = 0.68). Heterogeneity-based measures (18) provided only small gains relative to US (ΔAUC = +0.05). Two studies reported that SWE performed poorly in fatty or mixed lesions, with specificity as low as 26% (23).

**Table 3:** Paired comparative diagnostic accuracy summary (direct comparative studies only).

Author (year)	Comparison	Δ Sensitivity (SWE - comparator, %)	Δ Specificity (SWE - comparator, %)	Δ AUC (SWE - comparator)	Key direction of effect
16	SWE vs US	+15 (93 vs 78)	-10 (72 vs 82)	+0.07 (0.87 vs 0.80)	SWE > US (in benign subset)
17	SWE vs US	-4 (79 vs 83)	-5 (80 vs 85)	n/a	No difference
19 †	SWE vs US	-30 (50 vs 80)	+5 (79 vs 74)	-0.20 (0.65 vs 0.85)	SWE < US
18	rtSWE vs US	-5 (67 vs 71)	-2 (85 vs 87)	−0.05 (0.80 vs 0.85)	rtSWE slightly lower than US; complementary heterogeneity metric
24 (a)	ARFI SWE vs US	0 (77 vs 77)	0 (79 vs 79)	n/a	No difference
21	SWE (MSV) vs MRI	-10 (84 vs 94)	-9 (70 vs 79)	-0.06 (0.84 vs 0.90)	MRI > SWE
23 †	SWE vs US/MRI	+2 (83 vs 81)	-56 (26 vs 82)	-0.25 (0.57 vs 0.82-0.89)	SWE < US/MRI

†Non-paired comparison: Comparator values drawn from the same publication’s reference ultrasound or MRI cohort; included for directional context only.

Δ values, (SWE - Comparator). Positive Δ, SWE higher; negative Δ, SWE lower.

AUC, area under the receiver-operating-characteristic curve; SMI, superb microvascular imaging; ARFI, acoustic radiation force impulse; MSV, mean shear velocity.

MRI consistently showed higher discrimination than SWE (21: MRI AUC 0.90 vs SWE 0.84). Across modalities, changes in sensitivity ranged from −30% to +15%, specificity from −56% to +5%, and AUC differences from −0.25 to +0.07 (**Table 3**).

**Figure 4:**
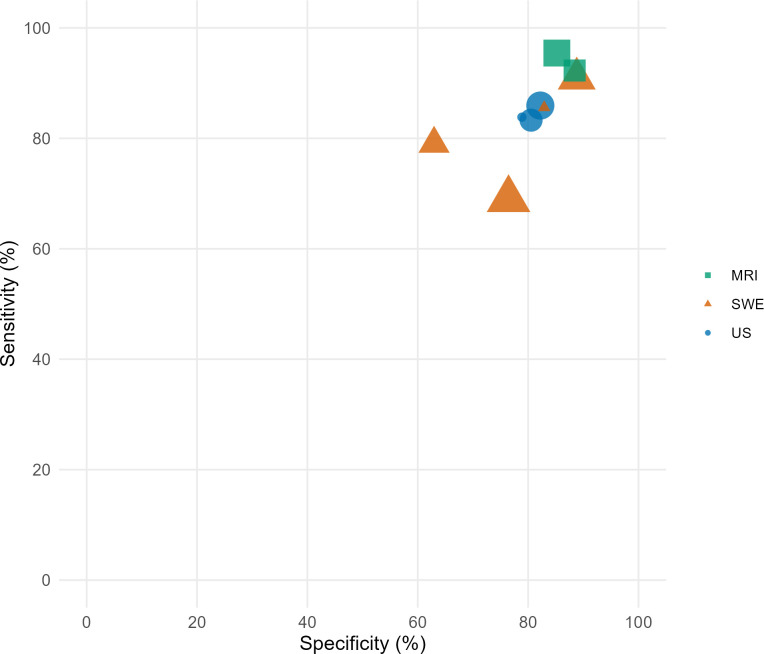
Area-under-the-curve (AUC) values for US, SWE, and MRI across comparative studies. Forest-style plot displaying reported AUCs by modality for each study.

**Figure 5:**
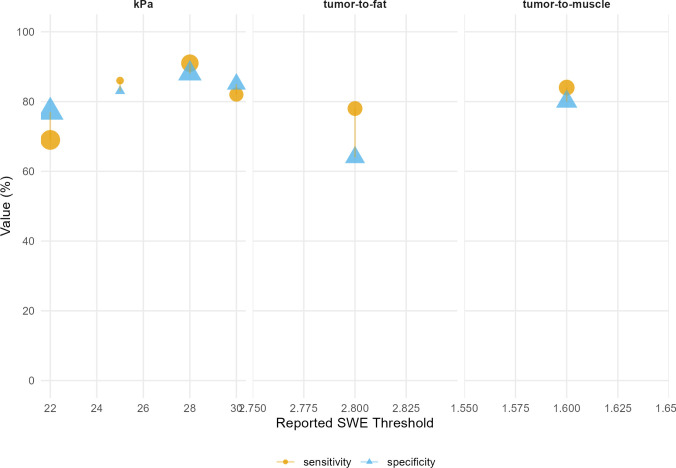
Reported shear-wave elastography (SWE) thresholds and corresponding diagnostic accuracy. Bubble plot showing sensitivity and specificity associated with reported SWE thresholds across studies, including absolute stiffness (kPa) and ratio-based cut-offs (tumor-to-fat, tumor-to-muscle).

**Figures 3**–**5** visualize these patterns.

**Figure 3** illustrates sensitivity-specificity dispersion, with SWE ranging 50-93% sensitivity and 26-85% specificity, compared with tighter clustering for US and MRI.

**Figure 4** plots AUC values, confirming SWE’s wider variability (0.57-0.87) relative to US (0.80-0.85) and MRI (0.85-0.90).

**Figure 5** summarizes reported SWE thresholds across absolute (kPa) and ratio-based metrics; thresholds between 24–30 kPa produced moderate performance, whereas ratio measures showed inconsistent accuracy. No uniform SWE cutoff generalized across studies.

Diagnostic reliability declined for deep (≥ 3 cm) or large (≥ 5 cm) masses, where MRI consistently outperformed both SWE and US (17). Context-dependent diagnostic performance across lesion subtypes is summarized in **Table 4**. For lipomatous tumors, SWE failed to distinguish lipoma from well-differentiated liposarcoma (23), while for solid or vascular lesions, elasticity heterogeneity enhanced discrimination (17, 18). Regarding the two studies that showed minimal stiffness separation: Ozturk et al. (22) reported mean pSWE velocities of 3.3 m/s for malignant lesions versus 3.0 m/s for benign lesions (p = 0.31; 95% CI overlapping across groups), suggesting that point-SWE offered no discriminatory value in their mixed population, likely attributable to a high proportion of deep intramuscular lipomatous tumors and the use of VTIQ technology without depth correction. Ooi et al. (23) similarly reported near-identical mean stiffness values for lipoma and well-differentiated liposarcoma (median AUC 0.57; pre-specified threshold range 1.78-2.02 m/s; p = 0.18 for stiffness between groups), with the major confounders identified as the high proportion of lipomatous lesions (estimated ~45% of the cohort) and poor cross-vendor measurement agreement (ICC 0.62).

**Table 4:** Context-dependent diagnostic performance of ultrasonography (US), shear-wave elastography (SWE), and magnetic resonance imaging (MRI) in soft-tissue tumors.

Lesion context	US	SWE	MRI	Key supporting studies (author + year) and notes
Superficial (< 3 cm)	●●●●○	●●●●●	●●●●●	(16) - SWE improves discrimination for superficial benign/indeterminate lesions; (18) - E_sd heterogeneity useful; (21) - combined US + SWE + vascularity increases accuracy.
Deep (≥ 3 cm)	●●○○○	●●●○○	●●●●●	(16) - inverse SWV-malignancy relation; (17) - depth reduces SWV; MRI consistently superior for intramuscular lesions.
Small (< 3 cm Ø)	●●●○○	●●●●○	●●●●○	(16) & (18) - SWE adds diagnostic value when US appearance benign; MRI and SWE comparable.
Large (≥ 5 cm Ø)	●●○○○	●●●○○	●●●●●	(17) - size and vascularity dominate; MRI essential for staging and extent evaluation.
Lipomatous	●●●●○	●●○○○	●●●●●	(17) & (23) - SWE fails to separate lipoma vs WDL/ALT; MRI + biopsy (MDM2) remains reference.
Solid/vascular	●●●○○	●●●●●	●●●●●	(18) - SWE heterogeneity (E_sd) improves differentiation; (17) - SWE highlights vascular benign lesions; MRI strong overall.

●●●●●, Excellent ●●●●○, Good ●●●○○, Moderate ●○○○○, Poor (Ratings based on median sensitivity/specificity/AUC ranges and study conclusions..).

**Figure 6:**
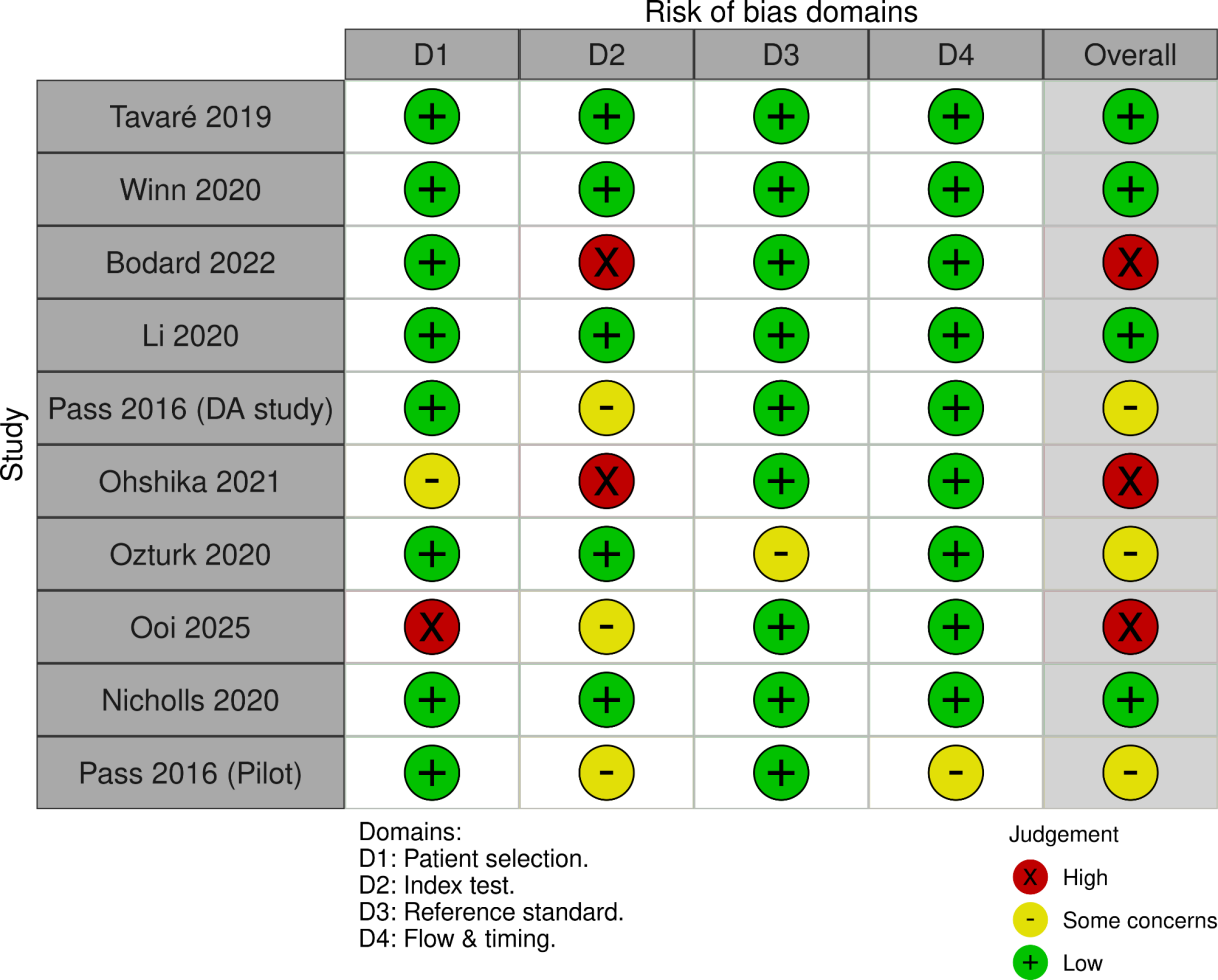
Risk of bias assessment of included studies using QUADAS-2 domains.

The risk of bias assessment (**Figure 6**) showed that most studies demonstrated low or acceptable methodological quality across the QUADAS-2 domains. The majority were judged low risk for patient selection (D1) and reference standard (D3). Certainty of evidence ratings based on the GRADE-DTA approach are summarized in **Supplementary Table 1**. Certainty for SWE versus US in superficial, non-lipomatous lesions was rated Moderate (⊕⊕⊕○), supported by consistent directionality in specificity and negative predictive value across studies. Although marked threshold heterogeneity was present across all ten included studies, the Inconsistency domain was specifically not downgraded for the superficial, non-fatty subgroup because three studies (16, 18, 21) showed directionally coherent AUC improvements and consistently higher specificity with SWE versus US alone (Sp range 72-85%). The Imprecision domain was downgraded by one level based on the pre-specified surrogate criteria (small subgroup size; sensitivity range exceeding 20 percentage points). Risk of bias was not downgraded as all three qualifying studies met the ≥90% histologic verification threshold, and Indirectness was not downgraded as study populations were representative of sarcoma referral centers. With only one downgrade trigger active, the rating therefore remained Moderate rather than Low. In contrast, for deep and lipomatous lesions, both Inconsistency and Imprecision were triggered, yielding a Low certainty rating (⊕⊕○○), consistent with the marked heterogeneity and limited data observed in those subgroups.

## Discussion

In this systematic review of ten studies evaluating shear-wave elastography (SWE) for extra-osseous soft-tissue masses, we found that although SWE demonstrated good reproducibility in most studies (inter-class correlation coefficient > 0.85), its diagnostic accuracy for differentiating benign from malignant lesions was inconsistent and generally inferior to magnetic resonance imaging (MRI). In the largest cohort, SWE offered only modest additional discrimination over conventional ultrasound (US) for lesions initially appearing benign (AUC 0.87) and still performed below MRI (AUC 0.89) (16). Other studies, including those by, Winn et al. (17), and Bodard et al. (19), likewise reported moderate specificity but low sensitivity, particularly in heterogeneous or deep lesions. Overall, these findings indicate that SWE may serve as a useful adjunct in superficial, benign-appearing masses but is not an adequate substitute for MRI in high-risk or complex tumors.

A consistent pattern across studies was the context-dependent performance of SWE. In superficial, small (< 3 cm), non-fatty lesions, SWE provided meaningful incremental value. Li et al. (18), for example, reported that the heterogeneity metric E_SD > 0.8 m/s was the strongest SWE predictor (AUC 0.80) and slightly improved the AUC of US alone (0.85). In contrast, performance declined markedly in deeper or lipomatous lesions. Bodard et al. (19) found sensitivity of only 50 percent and AUC 0.65, with substantial variation by lesion subtype. Similarly, Ooi et al. (23) reported that SWE could not distinguish lipoma from well-differentiated liposarcoma (AUROC 0.57) and showed poor cross-vendor reproducibility. These results highlight the influence of lesion depth, tissue composition, and platform-specific factors on SWE accuracy.

The broader literature reinforces this interpretation of SWE as an adjunctive rather than stand-alone technique. Cipriano et al. (14), in a scoping review of musculoskeletal SWE, identified considerable methodological heterogeneity across platforms, ROI strategies, depth parameters, and heterogeneity metrics, resulting in inconsistent diagnostic findings. Snoj (26) similarly emphasized that stiffness values are affected by anisotropy, pre-load, lesion depth, and surrounding tissue properties, limiting the interpretability of SWE in isolation. Thus, the patterns observed in our review align with wider experience: SWE shows promise but remains highly sensitive to technical and clinical context.

Mechanistically, the rationale for SWE rests on the generally greater stiffness of malignant lesions arising from increased cellularity, extracellular matrix disorganization, and fibrosis. This principle is well described in elastography literature across breast, thyroid, liver, and lymph-node imaging (27–30). Demonstrated that combining SWE with B-mode ultrasound significantly improved specificity for breast lesions while preserving high sensitivity. Although these findings provide a sound theoretical basis for SWE in soft-tissue tumor evaluation, their translation to musculoskeletal settings is complicated by deep lesion location, heterogeneous histologies, anisotropy, and vendor-related variability.

Our review therefore identifies several methodological and technical factors that likely explain the variable performance of SWE across studies.

### Depth and anisotropy effects

Several studies (17, 25) showed that shear-wave velocity decreased with increasing lesion depth or size (r = −0.40 in one series). Depth-related attenuation and increased heterogeneity of larger lesions may reduce the accuracy of SWE. This is consistent with prior musculoskeletal elastography literature showing that shear-wave velocity may underestimate stiffness in deeper tissues or in tissues with anisotropic structure (26).

### Vendor/platform heterogeneity and calibration

(23) found only moderate cross-vendor ICC (0.62 for velocity between machines) and markedly different diagnostic accuracy by platform. Technical literature confirms that shear-wave speed estimations are sensitive to beam-forming assumptions, speed-of-sound corrections, and pre-compression effects. For example, (31) demonstrated that constant speed-of-sound beamforming may introduce bias in shear-wave speed estimation. This suggests that threshold values derived on one machine may not generalize to another, limiting the external validity of SWE cut-offs in soft-tissue tumor imaging.

### Threshold heterogeneity

Across studies, threshold values for classifying malignant vs benign lesions ranged widely (in our data, e.g., 40.8 kPa in Bodard et al.; 1.78-2.02 m/s in Tavaré et al.). This variation undermines the feasibility of a universal cut-off. The methods we reviewed indeed identified substantial threshold heterogeneity (as defined by [max − min]/mean > 50%) which prevented meaningful pooling of fixed thresholds. The broader elastography literature also warns that variability in acquisition technique, ROI size/placement, and lesion morphology make universal thresholds risky (32).

### Heterogeneous histologic spectrum and case-mix

Soft-tissue tumors include a broad spectrum of benign and malignant histologies with differing mechanical properties. In our review, SWE discriminated non-fatty lesions more effectively, whereas lipomatous tumors remained a major limitation. Benign lipomas and low-grade liposarcomas often display overlapping stiffness values, which limits the utility of SWE in this subgroup (14, 23).

Given these constraints, SWE appears most useful as a supplementary tool for superficial, small (< 3 cm), non-fatty masses evaluated under standardized protocols. In these contexts, SWE may increase confidence in classifying benign-appearing lesions and modestly reduce unnecessary MRI or biopsy. For instance, Tavaré et al. (16) reported a 0.07 improvement in AUC when SWE was added to US in benign-appearing lesions. For deep, large, heterogeneous, or lipomatous lesions, MRI remains the superior modality for extent, tissue characterization, and malignancy prediction. Our findings reaffirm that MRI consistently achieves higher AUCs than SWE or US, supporting its continued role in diagnostic pathways where malignancy risk is higher.

The substantial vendor and platform variability further reinforces the need for machine-specific thresholds. Centers should validate local cutoffs, calibrate systems, and report indeterminate SWE measurements rather than forcing classification. Consistent ROI placement and documented acquisition parameters are essential for reliable interpretation.

SWE is most effective when integrated into a multiparametric ultrasound workflow. One study combining SMI vascularity with SWE achieved an AUC of 0.90, and similar advantages have been demonstrated in lymph-node imaging, where multiparametric approaches outperform B-mode alone (33). These findings indicate that SWE should complement, not replace, other imaging features within a broader diagnostic algorithm (34).

### Future research priorities

To increase the evidence quality and enable meta-analytic pooling, future studies should (i) use standardized SWE acquisition protocols and report key details (e.g., depth, ROI size, pre-compression, anisotropy), (ii) include consecutive cohorts with ≥ 90% histologic verification of all lesions (to minimize verification bias), (iii) report full 2×2 data including indeterminate SWE measurements, (iv) explore vendor-specific calibration and provide cross-platform comparisons, and (v) stratify by lesion type, depth, size and composition (fatty vs non-fatty). Only such standardization will permit robust threshold-setting and reliable risk-stratified recommendations.

Finally, regarding the appropriateness of a narrative synthesis: Although our original methods planned bivariate random-effects meta-analysis, the marked threshold heterogeneity, variable reporting of 2×2 tables, and low number of comparable studies precluded reliable pooling of fixed thresholds and summary sensitivity/specificity. This is consistent with the findings of the scoping review (14), which concluded that heterogeneity in musculoskeletal SWE studies was too high to support reliable meta-analysis.

### Implications for practice

- SWE is best used as an adjunct to ultrasonography, not a replacement for MRI or biopsy.
- Its clearest value is in superficial, small, non-fatty lesions that appear benign or indeterminate on B-mode/Doppler.
- SWE should not be used to differentiate lipoma vs ALT/WDL, or to risk-stratify deep or large lesions.
- Suspicious US features should override SWE; discordant or limited SWE results should prompt MRI.
- Use standardized protocols (probe, ROI placement, depth, number of measurements) and validate local thresholds.
- Training should focus on recognizing artifacts and depth attenuation, and reports should document acquisition parameters.
- SWE may reduce unnecessary MRI referrals in low-risk superficial masses, but offers little value in lipomatous or deep lesions.
- Guidelines should position SWE as an optional adjunct within an ultrasound-first pathway for appropriate superficial lesions.

### Limitations

This review has several limitations that mirror those of the included studies. First, heterogeneity in study design, lesion composition, and SWE acquisition parameters precluded formal bivariate meta-analysis. Instead, descriptive synthesis was performed using median and interquartile ranges, as prespecified in the protocol, acknowledging the statistical instability of pooled estimates under extreme threshold variability. Second, several included studies used single-center cohorts with expert radiologists, potentially inflating performance relative to general practice. Third, publication bias may persist, as studies with non-significant SWE findings are less likely to be published. Fourth, the search was restricted to English-language publications, which introduces potential language bias; studies reporting predominantly negative or null SWE findings published in other languages may have been missed, possibly overestimating the overall diagnostic utility of SWE. Finally, interobserver reliability, while high in controlled settings, has not been well studied across operators with different levels of experience.

## Conclusions

SWE offers reproducible, non-invasive quantification of tissue stiffness and holds potential as an adjunct to conventional ultrasonography in the evaluation of soft-tissue masses. However, its diagnostic accuracy is inconsistent across studies, influenced by lesion depth, composition, and vendor calibration differences. MRI remains the superior modality for comprehensive lesion characterization. Until standardization and multicenter validation are achieved, SWE should be interpreted cautiously as an informative but not definitive component of multimodal musculoskeletal imaging.

## Data Availability

The datasets presented in this study can be found in online repositories. The names of the repository/repositories and accession number(s) can be found in the article/**Supplementary Material**.
