# Supplementary material for: Diagnostic performance and clinical utility of shear-wave elastography in musculoskeletal soft-tissue tumors: a systematic review
Source: Front Oncol. 2026 Jun 30;16:1768309. doi: 10.3389/fonc.2026.1768309 (PMC13364610; doi:10.3389/fonc.2026.1768309)
Supplement: Supplementary file 1 [file DataSheet1.docx]

**PubMed (MEDLINE)**

(

"Elasticity Imaging Techniques"[Mesh]

OR elastograph*[tiab]

OR "shear wave elastograph*"[tiab]

OR "shear-wave elastograph*"[tiab]

OR "shearwave elastograph*"[tiab]

OR SWE[tiab]

OR "acoustic radiation force impulse"[tiab]

OR ARFI[tiab]

OR "point shear wave"[tiab]

OR pSWE[tiab]

OR "2D shear wave"[tiab]

OR "two-dimensional shear wave"[tiab]

OR "supersonic shear imaging"[tiab]

OR SSI[tiab]

)

AND

(

"Soft Tissue Neoplasms"[Mesh]

OR "Sarcoma"[Mesh]

OR ("soft tissue"[tiab] AND (tumor*[tiab] OR tumour*[tiab] OR neoplasm*[tiab] OR mass*[tiab] OR lesion*[tiab]))

OR ("musculoskeletal"[tiab] AND (tumor*[tiab] OR tumour*[tiab] OR neoplasm*[tiab] OR mass*[tiab] OR lesion*[tiab]))

OR "soft tissue sarcoma*"[tiab]

)

AND

(

ultrason*[tiab] OR ultrasound*[tiab] OR sonograph*[tiab]

OR "Ultrasonography"[Mesh]

)

**Embase**

(

'shear wave elastography'/exp

OR 'elastography'/exp

OR (elastograph* OR "shear wave elastograph*" OR "shear-wave elastograph*" OR SWE OR "acoustic radiation force impulse" OR ARFI OR "point shear wave" OR pSWE OR "2D shear wave" OR "two-dimensional shear wave" OR "supersonic shear imaging" OR SSI):ti,ab,kw

)

AND

(

'soft tissue tumor'/exp

OR 'soft tissue neoplasm'/exp

OR 'soft tissue sarcoma'/exp

OR 'sarcoma'/exp

OR (("soft tissue" OR musculoskeletal) NEAR/3 (tumor* OR tumour* OR neoplasm* OR mass* OR lesion*)):ti,ab,kw

)

AND

(

'ultrasonography'/exp

OR (ultrasound* OR ultrasonograph* OR sonograph*):ti,ab,kw

)

**Web of Science Core Collection**

TS=(

("shear wave" NEAR/2 elastograph*)

OR ("shear-wave" NEAR/2 elastograph*)

OR elastograph*

OR SWE

OR "acoustic radiation force impulse"

OR ARFI

OR ("point" NEAR/2 "shear wave")

OR pSWE

OR ("2D" NEAR/2 "shear wave")

OR ("two-dimensional" NEAR/2 "shear wave")

OR "supersonic shear imaging"

OR SSI

)

AND TS=(

("soft tissue" NEAR/2 (tumor* OR tumour* OR neoplasm* OR mass* OR lesion*))

OR ("musculoskeletal" NEAR/2 (tumor* OR tumour* OR neoplasm* OR mass* OR lesion*))

OR ("soft tissue" NEAR/2 sarcoma*)

OR "soft tissue sarcoma*"

)

AND TS=(ultrasound* OR ultrasonograph* OR sonograph*)

**Scopus**

TITLE-ABS-KEY(

elastograph* OR "shear wave elastograph*" OR "shear-wave elastograph*" OR SWE

OR "acoustic radiation force impulse" OR ARFI OR "point shear wave" OR pSWE

OR "2D shear wave" OR "two-dimensional shear wave"

OR "supersonic shear imaging" OR SSI

)

AND TITLE-ABS-KEY(

("soft tissue" W/3 (tumor* OR tumour* OR neoplasm* OR mass* OR lesion*))

OR ("musculoskeletal" W/3 (tumor* OR tumour* OR neoplasm* OR mass* OR lesion*))

OR "soft tissue sarcoma*" OR sarcoma*

)

AND TITLE-ABS-KEY(ultrasound* OR ultrasonograph* OR sonograph*)

**ProQuest Dissertations and Theses**

(

elastograph* OR "shear wave elastograph*" OR "shear-wave elastograph*" OR SWE

OR "acoustic radiation force impulse" OR ARFI OR "point shear wave" OR pSWE

OR "2D shear wave" OR "two-dimensional shear wave"

OR "supersonic shear imaging" OR SSI

)

AND

(

("soft tissue" NEAR/3 (tumor* OR tumour* OR neoplasm* OR mass* OR lesion*))

OR ("musculoskeletal" NEAR/3 (tumor* OR tumour* OR neoplasm* OR mass* OR lesion*))

OR "soft tissue sarcoma*" OR sarcoma*

)

AND

(ultrasound* OR ultrasonograph* OR sonograph*)

**RSNA meeting abstracts and proceedings**

("shear wave elastography" OR "shear-wave elastography" OR SWE OR ARFI OR "acoustic radiation force impulse" OR "supersonic shear imaging")

AND

("soft tissue" OR musculoskeletal OR extremit* OR trunk OR "soft tissue tumor" OR "soft tissue tumour" OR sarcoma OR "soft tissue mass")

**WFUMB abstracts and proceedings**

("shear wave elastography" OR "shear-wave elastography" OR SWE OR ARFI OR "acoustic radiation force impulse")

AND

("soft tissue" OR musculoskeletal OR "soft tissue tumor" OR "soft tissue tumour" OR sarcoma OR "soft tissue mass")
